# Multiplex ligation-dependent probe amplification assay identifies additional copy number changes compared with R-band karyotype and provide more accuracy prognostic information in myelodysplastic syndromes

**DOI:** 10.18632/oncotarget.13688

**Published:** 2016-11-29

**Authors:** Jingya Wang, Xiaofei Ai, Tiejun Qin, Zefeng Xu, Yue Zhang, Jinqin Liu, Bing Li, Liwei Fang, Hongli Zhang, Lijuan Pan, Naibo Hu, Shiqiang Qu, Wenyu Cai, Kun Ru, Yujiao Jia, Gang Huang, Zhijian Xiao

**Affiliations:** ^1^ State Key Laboratory of Experimental Hematology, Institute of Hematology and Blood Diseases Hospital, Chinese Academy of Medical Sciences & Peking Union Medical College, Tianjin, China; ^2^ Department of Pathology, Institute of Hematology and Blood Diseases Hospital, Chinese Academy of Medical Sciences & Peking Union Medical College, Tianjin, China; ^3^ MDS and MPN Centre, Institute of Hematology and Blood Diseases Hospital, Chinese Academy of Medical Sciences & Peking Union Medical College, Tianjin, China; ^4^ Divisions of Experimental Hematology and Cancer Biology, Cincinnati Children's Hospital Medical Center, Cincinnati, Ohio, USA

**Keywords:** myelodysplastic syndromes, cytogenetic analysis, multiplex ligation-dependent probe amplification

## Abstract

Cytogenetic analysis provides important diagnostic and prognostic information for patients with Myelodysplastic syndromes (MDS) and plays an essential role in the International Prognostic Scoring System (IPSS) and the revised International Prognostic Scoring System (IPSS-R). Multiplex ligation-dependent probe amplification (MLPA) assay is a recently developed technique to identify targeted cytogenetic aberrations in MDS patients. In the present study, we evaluated the results obtained using an MLPA assay in 437 patients with MDS to determine the efficacy of MLPA analysis. Using R-banding karyotyping, 45% (197/437) of MDS patients had chromosomal abnormalities, whereas MLPA analysis detected that 35% (153/437) of MDS cases contained at least one copy-number variations (CNVs) .2/5 individuals (40%) with R-band karyotype failures had trisomy 8 detected using only MLPA. Clonal cytogenetic abnormalities were detected in 20/235 (8.5%) MDS patients with a normal R-band karyotype, and 12/20 (60%) of those patients were reclassified into a higher-risk IPSS-R prognostic category. When sequencing and cytogenetics were combined, the fraction of patients with MDS-related oncogenic lesions increased to 87.3% (233/267 cases). MLPA analysis determined that the median OS of patients with a normal karyotype (n=218) was 65 months compared with 27 months in cases with an aberrant karyotype (P=0.002) in 240 patients with normal or failed karyotypes by R-banding karyotyping. The high-resolution MPLA assay is an efficient and reliable method that can be used in conjunction with R-band karyotyping to detect chromosomal abnormalities in patients with suspected MDS. MLPA may also provide more accurate prognostic information.

## INTRODUCTION

Myelodysplastic syndromes (MDS) are a group of clonal stem cell disorders characterized by cytopenias, dysplasia in one or more cell lineages and ineffective hematopoiesis. MDS are associated with significant morbidity and mortality due to bone marrow failure or evolution to acute myeloid leukemia [1]. Cytogenetic analysis provides important diagnostic and prognostic information for patients with MDS and plays an essential role in the International Prognostic Scoring System (IPSS) and the revised International Prognostic Scoring System (IPSS-R)[2–3]. Common cytogenetic abnormalities detected at diagnosis include -7/7q-, -5/5q-, +8, 20q-, -Y, i(17q) or t(17p), -13/13q-, 11q-, 12p- or t(12p), and the most common abnormalities (-7/7q-, -5/5q-, +8 and 20q-) occur in approximately 40% of all MDS cases[4–7]. The gold standard of cytogenetic diagnostics for MDS remains conventional chromosome banding analysis of bone marrow metaphases. Fluorescence *in situ* hybridization (FISH) is increasingly being used for cytogenetic analysis because of its higher resolution and greater success rate [8]. However, FISH probes are costly and have relatively low resolution (~20kb at best); generally, only larger and more common lesions are detected using FISH.

Multiplex ligation-dependent probe amplification (MLPA) assay is a recently developed technique to identify targeted copy-number variations (CNVs) in up to 50 different genomic regions simultaneously [9]. Small probes (~50-70nt) are directed at regions of interest in MDS or to reference regions that are generally not altered in MDS, providing greater resolution than FISH and bacterial artificial chromosome-based array-based comparative genomic hybridization (aCGH) and equivalent resolution to oligo-based aCGH [10–11]. In this study, we evaluated the results obtained using an MLPA assay in patients with MDS to determine the efficacy of MLPA analysis.

## RESULTS

### The frequency of cytogenetic abnormalities in MDS patients

Using R-banding karyotyping, about 45% (197/437) of MDS patients had chromosomal abnormalities, whereas MLPA analysis detected that 35% (153/437) of MDS cases contained at least one CNV (Supplementary Figure S1). The most common CNVs detected using MLPA included +8 (12.6%), 5q- (10.3%), -7/7q- (7.6%), 20q- (7.8%) and 17p- (4.6%). Overall, a total of 50% of MDS patients (219/437) had cytogenetic abnormalities detected with these two methods combined. The frequency of genetic lesions determined by R-band karyotype and MLPA was listed in Table 1.

**Table 1:** Frequency of genetic lesions determined by R-band karyotype and MLPA (N=437)

	R-band karyotype (%)	MLPA (%)
−5/5q-	41(9.38)	45(10.30)
−7/7q-	28(6.41)	33(7.55)
+8	53(12.13)	55(12.59)
+11q	3(0.69)	10(2.29)
12p-	14(3.20)	15(3.43)
17p-	10(2.29)	20(4.58)
20q-	32(7.32)	34(7.78)

### MLPA complements R-band karyotype

The 437 MDS patients were divided into three subgroups based on R-band karyotype results. Of the 197 cases with abnormal R-band karyotypes, MLPA analysis detected that 66.5% (131/197) of cases had at least one CNV. 33.5% cases (66/197) showed discrepancies between MLPA and R-band results. Among the discrepancies, 12 cases had complex karyotypes. 41 cases were attributed to a failure of MLPA probes in targeting the chromosomal abnormalities and 10 cases harbored small clones. 22/197 (11.2%) had additional CNVs detected by MLPA compared with R-band karyotype, and 8/22 (36.4%) of those patients were reclassified into a higher-risk IPSS-R prognostic category. 2/5 individuals (40%) with R-band karyotype failures had trisomy 8 detected using only MLPA. Using MLPA analysis, clonal cytogenetic abnormalities were detected in 20/235 (8.5%) MDS patients with a normal R-band karyotype, and12/20 (60%) of those patients were reclassified into a higher-risk IPSS-R prognostic category. All the additional detected aberrations by MLPA are summarized in Table 2.

**Table 2:** Cases with additional copy number changes identified by MLPA compared to R-band karyotype

Case	Age/gender	WHO 2008	Karyotype	Chromosomal abnormalities detected by MLPA	IPSS-R risk group	IPSS-R risk group by MLPA
164	58/M	RAEB-1	NR	+8	NR	Very high
90	39/M	RAEB-2	NR	+8	NR	Very high
72	59/F	RCMD	46,XX[1]	5q-	High	High
52	37/F	RAEB-1	46,XX[5]	−7	High	Very high
51	47/F	RCMD	46,XX[6]	−7	Intermediate	High
37	42/M	RAEB-1	46,XY[9]	7q-	High	High
184	63/M	RAEB-1	46,XY[14]	7q-	High	Very high
36	51/M	RCMD	46,XY[3]	7q-	Low	Intermediate
74	53/F	RAEB-2	46,XX[13]	+8	Very high	Very high
442	60/M	RAEB-2	46,XY[20]	11q+	High	Very high
212	37/F	RAEB-1	46,XX[18]	11q+	High	Very high
362	68/M	RCMD	46,XY[20]	12p-	Low	Low
227	77/M	RCMD	46,XY[20]	17p-	Low	Intermediate
44	32/M	RCMD	46,XY[20]	17p-	Low	Intermediate
18	50/M	RAEB-1	46,XY[20]	17q-	Intermediate	High
406	76/M	RCMD	46,XY[20]	−19	Low	Low
134	57/F	RCMD	46,XX[10]	20q-	Low	Low
218	42/M	RCMD	46,XY[20]	20q-	Low	Low
88	41/M	RAEB-2	46,XY[7]	+8/11q+	High	Very high
406	63/M	RAEB-2	46,XY[9]	+8/17p-	Very high	Very high
142	38/F	RCMD	46,XX[15]	5q-/17p−/−19	Low	Intermediate
141	62/M	RAEB-1	46,XY[14]	5q-/12p-/17p-/ -19/20q-	High	Very high
300	72/M	RAEB-1	45,XY,-Y[5]/46,XY[15]	17p-	Low	Intermediate
344	68/M	RAEB-2	47,XY,+9,i(17)(q10)[3]/ 46,XY,i(17)(q10)[17]/ 46,XY[2]	17p−/−19/20q-	High	Very high
40	39/M	RAEB-1	46,XY,del(5)(q23)[10]	5q-/17p-	Intermediate	Intermediate
150	73/M	RCMD	45,X,-Y[8]/46,XY[2]	5q-	Low	Intermediate
161	53/M	RAEB-2	47,XY,+8[20]	5q-/+8	Very high	High
277	40/F	RAEB-1	45,XX,-7[18]/46,XX[20]	−7/17p−/−19/20q-	Very high	Very high
225	28/M	MDS-U	47,XY,+8,14ps+[4]	+8/12p-/17p-	Intermediate	High
139	53/M	RCMD	48,XY,+8,+21[10]	−5q/+8	Intermediate	High
9	47/M	RCMD	47,XY,+8[2]/46,XY[18]	+8/11q+	Low	Low
94	47/M	RAEB-1	46,XY,del(20)(q12)[20]	11q+/20q-	High	High
115	52/M	RAEB-1	46,XY,+X,del(20)(q12)[2]/46,XY,del(20)(q12)[4]	7q-/20q-	High	Very high
190	71/F	RAEB-2	46,XX,5q-,12q-[5]	5q-/+8/+11q	Very high	Very high
320	66/F	RCMD	41-45,XX,inc[cp19]/ 46,XX[1]	5q-/7q-	High	High
32	44/M	RCMD	47,XY,-22,+mar1,+mar2[8]/46,XY[2]	+8	High	High
41	59/F	RAEB-2	45,X,-X,del(5)(q13q33),del(6)(q23),add(17)(p13),del(17)(q22)[9]/46,XX[1]	5q−/−7/12p-	Very high	Very high
54	60/M	RAEB-1	45,XY,add(1)(q44),del(5)(q13q33),-12,add(17)(p10)[2]/45,XY,del(5)(q13q33),-12,add(17)(p10)[10]/46,XY[8]	5q-/+8/11q+/ 12p-	Very high	Very high
113	62/M	RCMD	42-46,XY,-2,-4,-6,-11,-12,-13,-17,+3-4mar,inc[cp10]	5q−/−7/−17p	Very high	Very high
154	50/F	RAEB1	43-46,X,-X,-12,-15,-17,-20,+1-2mar,inc[cp4]/46,XX[6]	5q-/+8	Very high	Very high
233	54/M	RCMD	43,XY,-5,-9,-21[13]/ 46,XY[7]	−5/7q-/12p−/−19	High	High
236	35/M	RCMD	49,XY,+Y,+8,+9[4]/ 46,XY[10]	+8/−19/20q-	High	Very high
318	46/M	RCMD	44,XY,add(2)(p25),-3,-5[12]/46,XY[8]	−7	High	Very high

### Genetic abnormalities combined cytogenetics and targeted gene sequencing

We sequenced 112 genes across 267 MDS patients. In total, 202 of 267 (75.7%) patients had at least one oncogenic mutation, whereas cytogenetic studies and MLPA identified abnormalities in 49.8% of the 267 patients. 7 mutations were present in ≥5% of patients: U2AF1 (17.6%), TET2 (15.4%), ASXL1 (13.9%), SF3B1 (12.4%), TP53 (8.2%), RUNX1 (6.0%) and DNMT3A (5.2%). When sequencing and cytogenetics were combined, the fraction of patients with MDS-related oncogenic lesions increased to 87.3% (233/267 cases).

### Implications of cytogenetic aberrations detected by MLPA on overall survival

**Figure 1:**
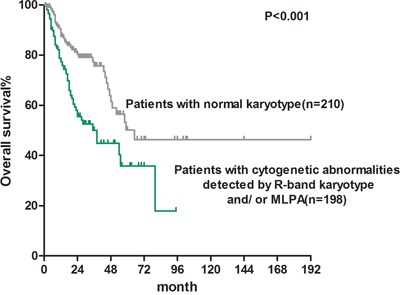
Overall survival of MDS patients with normal karyotype (median OS: 38 months) and patients without cytogenetic abnormalities detected by G-band karyotype and/ or MLPA (median OS: 65 months)

Patients with chromosomal abnormalities detected using R-band karyotyping and/or MLPA analysis had significantly shorter survival than patients with a normal karyotype {median overall survival (OS): 38 vs. 65 months, *P*<0.001; Figure 1}.

**Figure 2:**
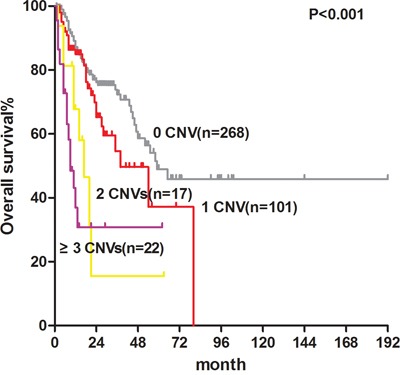
Overall survival of MDS patients with CNVs detected by MLPA: 0 CNV, 59months; 1 CNV, 54 months; 2 CNVs, 17 months; ≥3 CNVs, 9 months

According to the number of aberrations present in each case, as detected using MLPA, 408 cases were divided into 4 groups (0, 1, 2 and ≥3 CNVs), and the corresponding median OS of each group was 59, 54, 17 and 9 months (*P*<0.001; Figure 2), respectively.

### The impact of cytogenetic aberrations detected by MLPA on overall survival of higher-risk IPSS-R patients with a normal or failed karyotypes detected by R-banding

**Figure 3:**
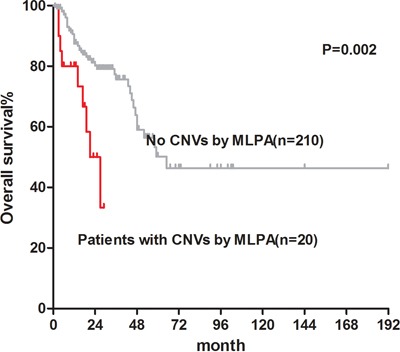
Overall survival of normal karyotype MDS patients with CNVs detected by MLPA (median OS: 27 months) and patients without CNVs (median OS: 65 months)

**Figure 4:**
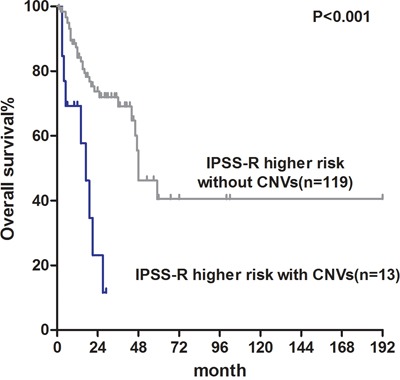
Overall survival of normal karyotype IPSS-R higher risk patients with CNVs detected by MLPA (median OS: 17 months) and patients without CNVs (median OS: 48 months)

We also performed survival analysis of 240 patients with normal or failed karyotypes. MLPA analysis determined that the median OS of patients with a normal karyotype (n=218) was 65 months compared with 27 months in cases with an aberrant karyotype (*P*=0.002; Figure 3). In lower-risk IPSS-R patients with a normal karyotype, 8% of cases (8/99) had cytogenetic aberrations detected by MLPA analysis. However, there were no differences in OS because no “poor” or “very poor” cytogenetic aberrations were detected in lower-risk patients (*P*=0.410; Supplementary Figure S2). In higher-risk IPSS-R patients with a normal karyotype, MLPA analysis revealed that 10% (14/141) of cases had cytogenetic aberrations. OS was significantly shorter in the 13 higher-risk patients with cytogenetic aberrations detected solely using MLPA compared with the other 119 higher-risk patients (median OS: 17 vs. 48 months, *P*<0.001; Figure 4).

## DISCUSSION

The development of MLPA analysis for multiple genetic loci has provided a new approach to routine diagnostic testing of CNVs in MDS patients. Owing to the high throughput capability of the technique, MLPA analysis kit can be updated rapidly according to the latest research progress in cytogenetic study. One of the major advantages of MLPA is its high specificity because this method is able to distinguish sequences that differ in length by only one nucleotide. Another advantage is the low amount of input DNA (minimum of 20-50 ng) required for a successful MLPA reaction [9]. However, balanced rearrangements, polyploidy and low proportions of cell clones (approximately 20%) are not identified using MLPA [15].

Studies have shown that MLPA has higher accuracy and specificity in the testing of acute myeloid leukemia and MDS than FISH [16]. In our study, we analyzed 437 MDS patients using an MLPA assay and detected clonal genetic abnormalities in 9.2% of cases with a normal or failed karyotype, which may alter the risk stratification of these patients. Furthermore, survival analysis demonstrated the adverse prognostic impact of CNVs in these patients. Volkert et al. [17] detected copy number changes in 11% of 520 MDS patients with a normal karyotype using array CGH. The proportion of patients with CNVs was lower in our cohort, which is most likely due to the targeted probes used in the MLPA analysis. Array CGH/ MLPA have been increasingly used as a method of choice for diagnosis of MDS patients with unexplained genetic aberrations.

High-throughput sequencing of 112 gene targets across 267 MDS patients, combined with copy number analyses, revealed a landscape of genetic lesions in MDS with a broad spectrum of gene mutations. Recurrently mutated genes typically occurred in six major gene pathways, including spliceosome genes, epigenetic modifiers, transcription factors, activated signaling/RAS, cohesin factors and TP53. Several groups have recently described mutations in candidate gene panels in *de novo* MDS [18–20], and over 80% of MDS samples harbored at least one mutated gene or a cytogenetic abnormality, which was confirmed by this study. Based on these findings we can improve the way to predict the prognosis of patients with MDS to enhance making clinical decisions.

In conclusion, the high-resolution MPLA assay is an efficient and reliable method that can be used in conjunction with R-band karyotyping to detect chromosomal abnormalities in patients with suspected MDS, and MLPA may provide more accurate prognostic information. Molecular alterations with possible prognostic value in MDS identified by MLPA and/ or NGS analyses serve as proofs of the disease and are supposed to be incorporated into the prognostic models.

## MATERIALS AND METHODS

### Patients

This study was approved by the Ethics Committee at the Institute of Hematology, the Chinese Academy of Medical Sciences and Peking Union Medical College, in accordance with the guidelines of the Declaration of Helsinki. A total of 437 patients were diagnosed with MDS according to the World Health Organization in 2008. Those classification were retrospectively enrolled between January 2001 and November 2015 (Table 3). Of these patients, 276 were males (63.2%), and 161 were females (36.8%). The median age of the patients was 51 years (range, 16 to 84 years). 432 (98.9%) subjects with evaluable cytogenetics were classified using the revised International Prognostic Scoring System (IPSS-R) criteria [3]. Because there is no official definition of lower- and higher-risk MDS in the IPSS-R system, we grouped the very-low-risk and low-risk IPSS-R groups into the lower-risk category and the intermediate-risk, high-risk and very-high-risk groups into the higher-risk category. 25 (5.7%) subjects received allotransplant, of the subjects receiving an allotransplants, all were censored in survival analyses. 120 (27.5%) subjects received erythropoietin with or without G-CSF, RBC and/or platelet transfusions and/or iron chelation with desferrioxamine. 112 (25.6%) subjects received immune suppressive drugs including cyclosporine and thalidomide [12]. 58 (13.3%) subjects received decitabine or Azacitidine, 34 subjects (5.5%) received anti-cancer therapy(ies) including aclacinomycin or homoharringtonine combined with cytarabine and granulocyte-colony stimulating factor (G-CSF; termed CAG or HAG), idarubicin or daunorubicin combined with cytarabine (IA or DA) or melphalan [13]. 11 (2.5%) subjects received traditional Chinese medicines. Survival data were available for 408(93.4%) patients; data were censored as of February 29, 2016 or at date of last contact, with a median follow-up of 16 months (range, 1-192months).

**Table 3:** Patients' characteristics (n=437)

	Total n (%)	Normal/failing karyotype n(%)	Abnormal karyotype n(%)
N. of patients	437 (100.0)	240 (54.9)	197 (45.1)
Median age (years) (range)	51 (16-84)	51 (16-84)	51 (16-82)
Sex			
Male	276 (63.2)	146 (60.8)	130 (66.3)
Median Hb (g/L) (range)	76 (28-173)	76 (28-173)	76 (35-153)
Median ANC (×10^9^/L) (range)	1.16 (0-20.53)	1.15 (0.09-20.53)	1.16 (0-13.21)
Median platelet count (×109/L) (range)	65 (2-1561)	63 (5-1024)	69 (2-1561)
WHO 2008			
RCUD	12 (2.7)	7 (2.9)	5 (2.5)
RARS	15 (3.4)	10 (4.2)	5 (2.5)
RCMD	231 (52.9)	135 (56.3)	96 (48.7)
RAEB-1	86 (19.7)	49 (20.4)	37 (18.8)
RAEB-2	77 (17.6)	36 (15.0)	41 (20.8)
MDS-U	11 (2.5)	3 (1.3)	8 (4.1)
5q- syndrome	5 (1.1)	0 (0.0)	5 (2.5)
IPSS risk category			
Low	39 (8.9)	32 (13.3)	7 (3.6)
Int-1	277 (63.4)	182 (75.8)	95 (48.2)
Int-2	95 (21.7)	26 (10.8)	69 (35.0)
High	26 (5.9)	0 (0.0)	26 (13.2)
IPSS-R risk category			
Very low	15 (3.4)	14 (5.8)	1 (0.5)
Low	116 (26.5)	85 (35.4)	31 (15.7)
Intermediate	138 (31.6)	84 (35.0)	54 (27.4)
High Very high	102 (23.3) 66 (15.1)	50 (20.8) 7 (2.9)	52 (26.4) 59 (29.9)

### Cytogenetic analysis

Karyotype analyses were performed on un-stimulated bone marrow cells after 24 h of culture using R-banding techniques [7]. Chromosome identification and karyotype descriptors used the International System for Human Cytogenetic Nomenclature (ISCN) [14].

### MLPA analysis

Bone marrow specimens were collected from subjects at diagnosis. Genomic DNA was extracted using the AxyPrep Blood Genomic DNA Miniprep Kit (Axygen Biosciences, cat no. AP-MN-BL-GDNA-250 Union city, CA, USA) and subjected to MLPA analysis using a SALSA MLPA P414-A1 Kit (MRC Holland, Amsterdam, Netherlands) according to the manufacturer's instructions. The probemix contained 46 probes targeted to chromosomal regions of interest in MDS (Supplementary Table S1) and 12 internal reference probes targeted to regions that are generally unchanged in MDS. Finally, PCR products were analyzed using an ABI 3730 capillary sequencer (Applied Biosystems, Foster City, CA USA) and Coffalyser software (MRC Holland, Amsterdam, Netherlands). To eliminate differences between the probes, a normal range for each MDS targeted probe was established to improve the accuracy of the MLPA analysis. In addition, 20 DNA samples derived from the peripheral blood of healthy donors were subjected to MLPA analysis. The “Mean±2SD” (95% CI, *P*=0.05) and “Mean±3SD” (95% CI, *P*=0.01) values for each individual probe are listed in Supplementary Table S1. To improve the evaluation of the results with a larger CI, the “Mean±3SD” reference range was used as the cutoff value for CNV determination in our study.

### Next generation sequencing (NGS) and mutation analysis

DNA was extracted from bone marrow mononuclear cells using the AxyPrep Blood Genomic DNA Miniprep Kit (Axygen Biosciences, cat no. AP-MN-BL-GDNA-250 Union city, CA, USA). A total of 267 MDS patients were detected by a custom targeted NGS gene panel designed covering 112 genes (Supplementary Table S2) associated with blood diseases. All exons of these genes were sequenced on an Ion Torrent semiconductor platform (ThermoFisher Scientific Inc, Waltham, Massachusetts) and reads were mapped to NCBI hg19 RefSeq. Validation runs showed accurate results, with a mean of >97% coverage of the targeted regions at the average depth of 800X. Polymorphisms annotated in dbSNP 135 were excluded. All sequencing data were analyzed using our in-house pipeline, through which high-probability oncogenic mutations were called by eliminating sequencing/ mapping errors and known/ possible SNPs based on the available databases and frequencies of variant reads.

### Statistical analysis

All statistical analyses considered clinical and laboratory parameters at diagnosis or first referral. Categorical variables are described as counts and relative frequencies (%). Survival was measured from date of diagnosis to death or last known of follow-up and estimated using the Kaplan-Meier method. The Log-rank test was used to compare survival data. All *P*-values were two-tailed, and statistical significance was set at *P*<0.05. Analyses were conducted using SPSS software, version 18.0. Data were censored as of February 29, 2016 or at date of last contact. Median follow up was 16 months (range, 1-192 months).

## SUPPLEMENTARY FIGURES AND TABLES




