# Supplementary material for: Multiplex ligation-dependent probe amplification assay identifies additional copy number changes compared with R-band karyotype and provide more accuracy prognostic information in myelodysplastic syndromes
Source: Oncotarget. 2016 Nov 29;8(1):1603–12. doi: 10.18632/oncotarget.13688 (PMC5352081; doi:10.18632/oncotarget.13688)
Supplement: Supplementary file 1 [file oncotarget-08-1603-s001.pdf]

Multiplex ligation-dependent probe amplification assay identifies additional copy number changes compared with R-band karyotype and provide more accuracy prognostic information in myelodysplastic syndromes

SUPPLEMENTARY FIGURES AND TABLES

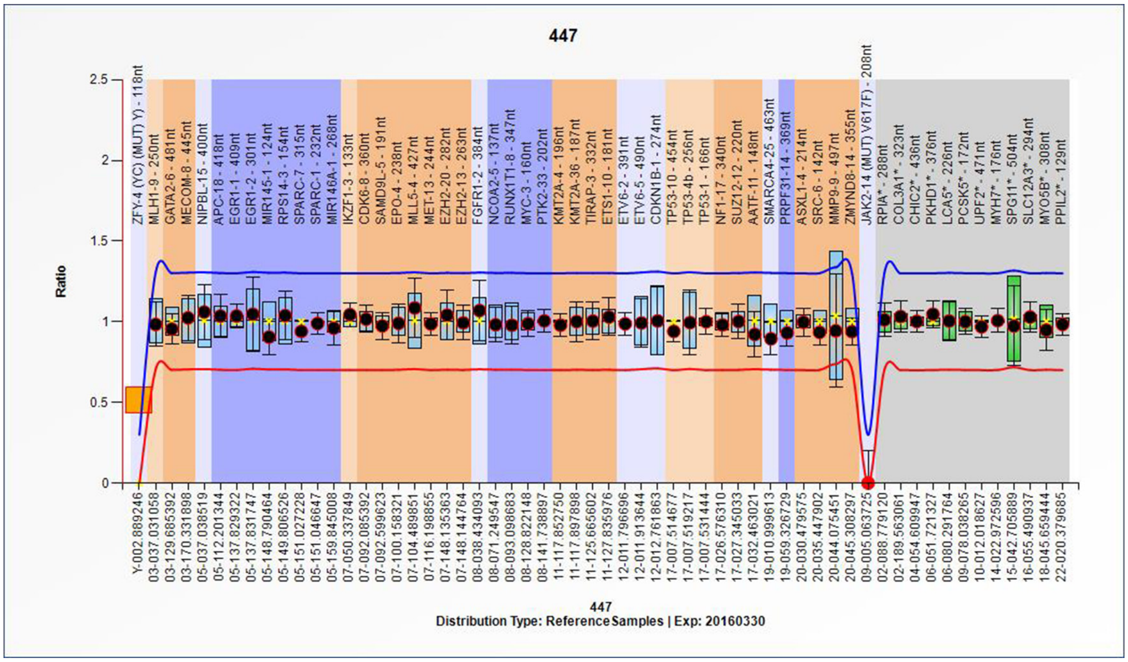

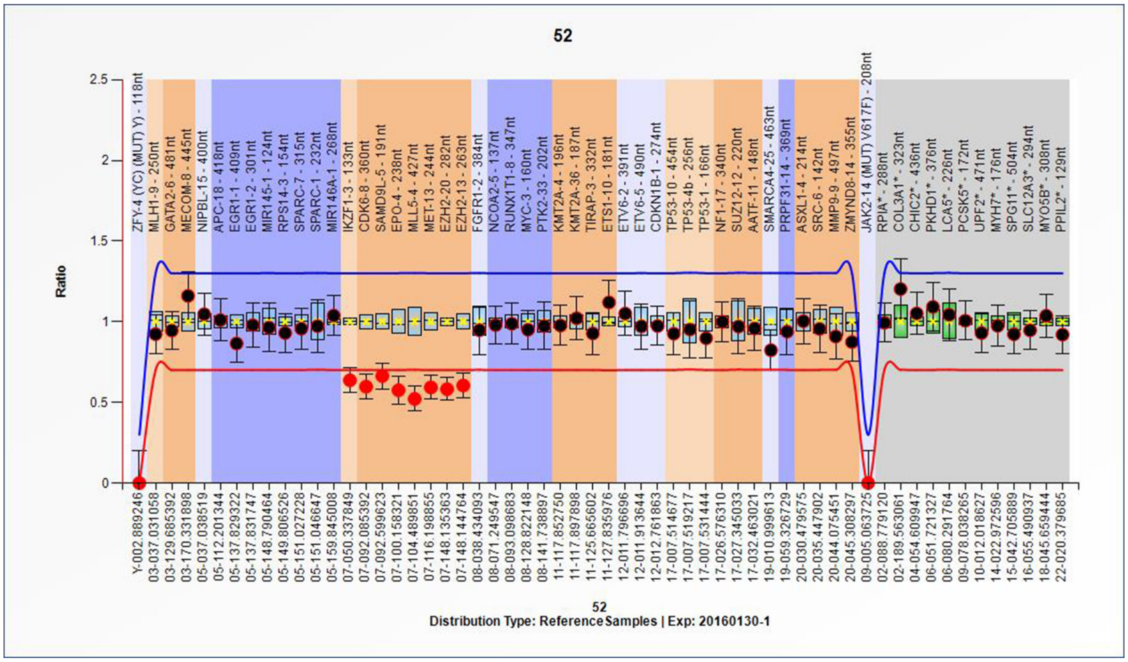

(*Continued*)

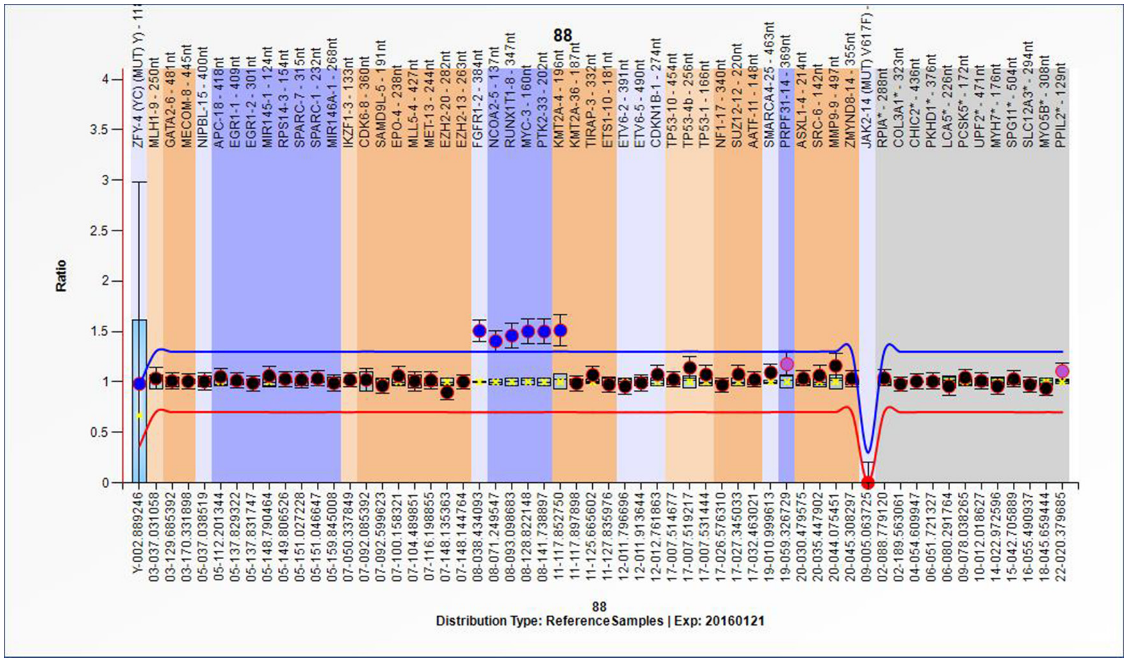

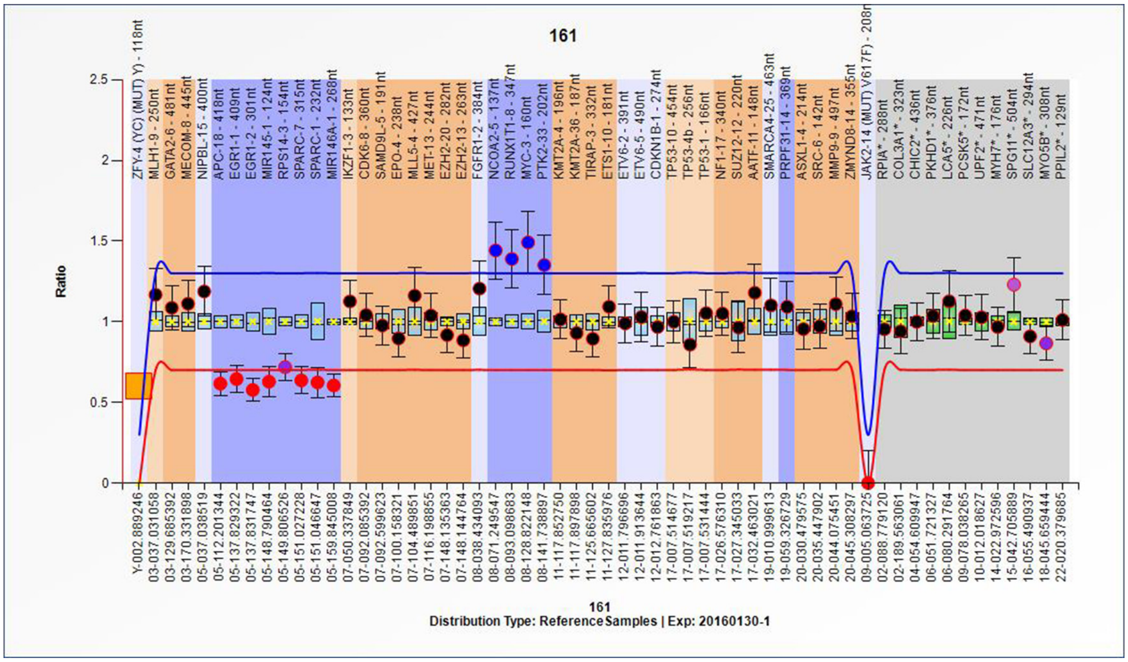

Supplementary Figure S1: The abnormalities detected by MLPA. A. Normal karyotype. **B.** MLPA detection of -7 in a patient with normal G-band karyotype. **C.** MLPA detection of +8/11q+ in a patient with normal G-band karyotype. **D.** MLPA detection of 5q-/+8 in a patient with 47,XY,+8[20].

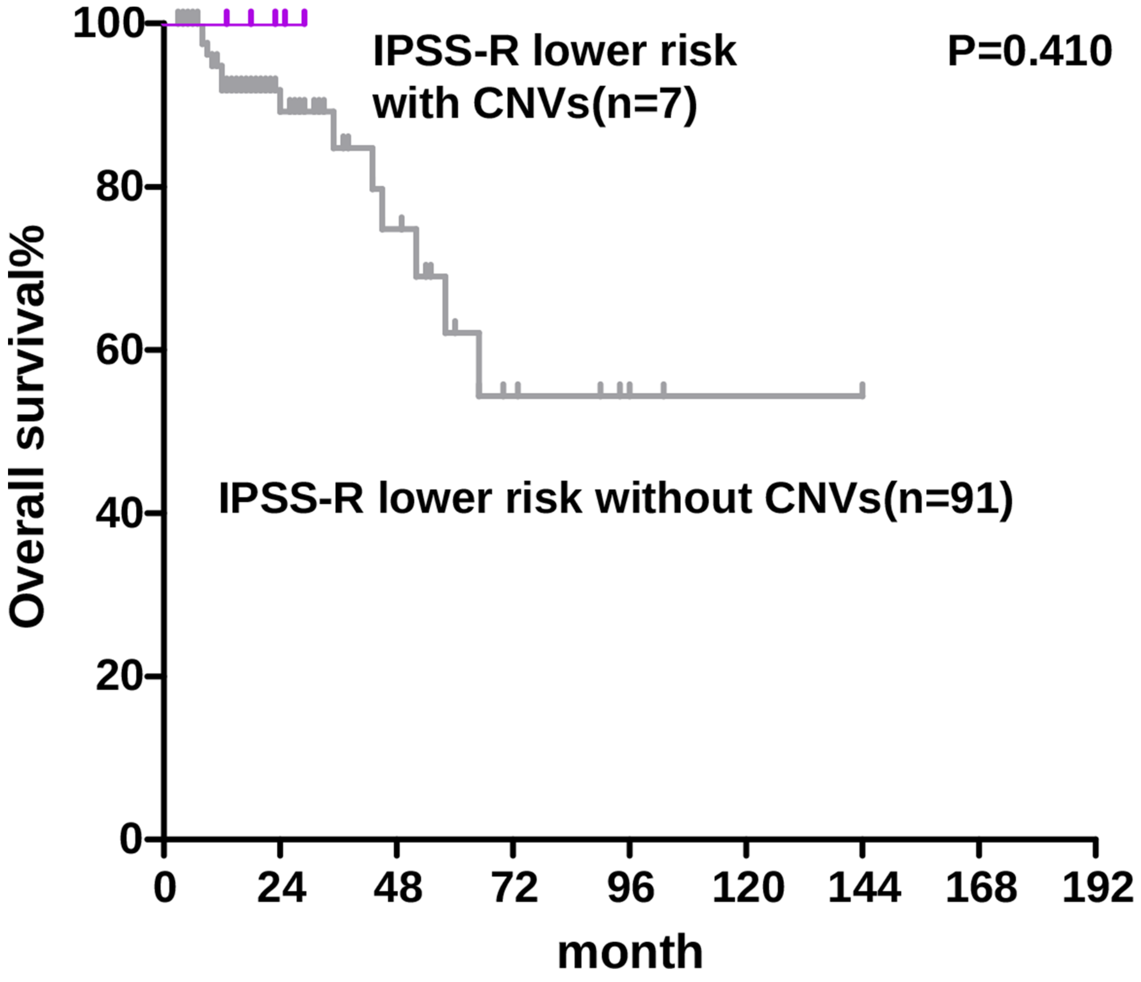

Supplementary Figure S2: Overall survival of normal karyotype IPSS-R lower risk patients with CNVs detected by MLPA (median OS: not reached) and patients without CNVs (median OS: not reached).

Supplementary Table S1: Probe-specific normal reference ranges established in multiplex ligation-dependent amplifications assay for MDS

See Supplementary File S1

Supplementary Table S2: The 112 genes sequenced in this study

ABCB1	ABL1	ADAMTS13	AKT1	ALAS2	ARID1A	ASXL1	ATM
BCL2	BCL6	BIRC3	BRAF	CALR	CBL	CCND1	CCND3
CDKN1A	CEBPA	c-MAF	c-MYC	CREBBP	CRLF2	CSF3R	CUX1
CXCR4	CYLD	DDX3X	DIS3	DNM2	DNMT3A	ECT2L	EED
EGFR	EP300	EPHA7	EZH2	FAM46C	FANCA	FANCC	FANCG
FAT1	FBXW7	FGFR3	FLT3	GATA2	GATA3	IDH1	IDH2
IL7R	ITK	JAK1	JAK2	JAK3	KIT	KRAS	SH2B3
LYST	MAFB	MAPK1	MLL2	MPL	MUM1	MYD88	MYH11
NF1	NOTCH1	NOTCH2	NPM1	NRAS	PAX5	PDGFRB	PHF6
PIK3CA	PRDM1	PRF1	PRMT5	PRPF40B	PTEN	PTPN11	RAB27A
RB1	RELN	RUNX1	SAMHD1	SETBP1	SF1	SF3A1	SF3B1
SH2D1A	SMC1A	SMC3	SRSF2	STX11	STXBP2	SUZ12	TAL1
TET2	TP53	TEL/ETV6	TRAF3	U2AF1	U2AF2	UNC13D	WAS
WHSC1	WT1	TNFAIP3/A20	XIAP	XPO1	ZMYM3	ZRSR2	IKZF1
