# Supplementary material for: Multiplex ligation-dependent probe amplification assay identifies additional copy number changes compared with R-band karyotype and provide more accuracy prognostic information in myelodysplastic syndromes
Source: Oncotarget. 2016 Nov 29;8(1):1603–12. doi: 10.18632/oncotarget.13688 (PMC5352081; doi:10.18632/oncotarget.13688)
Supplement: Supplementary file 2 [file oncotarget-08-1603-s002.docx]

Sup. Table1 Probe-specific normal reference ranges established in multiplex ligation-dependent amplifications assay for MDS.

| Target regions | Gene/Exon | Normal range(mean±2SD)  (95%CI, P<0.05) | Normal range(mean±3SD)  (99%CI, P<0.01) |
| --- | --- | --- | --- |
| Yp11.31 | ZFY-4 | 0.84-1.20 | 0.75-1.29 |
| 3p22.2 | MLH1-9 | 0.74-1.46 | 0.56-1.64 |
| 3q21.3 | GATA2-6 | 0.78-1.26 | 0.66-1.38 |
| 3q26.2 | MECOM-8 | 0.77-1.25 | 0.65-1.37 |
| 5p13.2 | NIPBL-15 | 0.76-1.44 | 0.59-1.61 |
| 5q22.2 | APC-18 | 0.94-1.06 | 0.91-1.09 |
| 5q31.2 | EGR1-1 | 0.93-1.09 | 0.89-1.13 |
| 5q31.2 | EGR1-2 | 0.87-1.15 | 0.80-1.22 |
| 5q32 | MIR145-1 | 0.85-1.17 | 0.77-1.25 |
| 5q33.1 | RPS14-3 | 0.90-1.10 | 0.85-1.15 |
| 5q33.1 | SPARC-7 | 0.86-1.18 | 0.78-1.26 |
| 5q33.1 | SPARC-1 | 0.82-1.22 | 0.72-1.32 |
| 5q34 | MIR146A-1 | 0.96-1.04 | 0.94-1.06 |
| 7p12.2 | IKZF1-3 | 0.77-1.13 | 0.68-1.22 |
| 7q21.2 | CDK6-8 | 0.76-1.36 | 0.61-1.51 |
| 7q21.2 | SAMD9L-5 | 0.94-1.06 | 0.91-1.09 |
| 7q22.1 | EPO-4 | 0.81-1.17 | 0.72-1.26 |
| 7q22.3 | MLL5-4 | 0.83-1.23 | 0.73-1.33 |
| 7q31.2 | MET-13 | 0.86-1.22 | 0.77-1.31 |
| 7q36.1 | EZH2-20 | 0.97-1.05 | 0.95-1.07 |
| 7q36.1 | EZH2-13 | 0.91-1.07 | 0.87-1.11 |
| 8p11.22 | FGFR1-2 | 0.82-1.26 | 0.71-1.37 |
| 8q13.3 | NCOA2-5 | 0.90-1.14 | 0.84-1.20 |
| 8q21.3 | RUNX1T1-8 | 0.84-1.28 | 0.73-1.39 |
| 8q24.21 | MYC-3 | 0.86-1.18 | 0.78-1.26 |
| 8q24.3 | PTK2-33 | 0.85-1.17 | 0.77-1.25 |
| 11q23.3 | KMT2A-4 | 0.90-1.10 | 0.85-1.15 |
| 11q23.3 | KMT2A-36 | 0.87-1.11 | 0.82-1.17 |
| 11q24.2 | TIRAP-3 | 0.84-1.20 | 0.75-1.29 |
| 11q24.3 | ETS1-10 | 0.92-1.12 | 0.87-1.17 |
| 12p13.2 | ETV6-2 | 0.88-1.12 | 0.82-1.18 |
| 12p13.2 | ETV6-5 | 0.91-1.11 | 0.86-1.16 |
| 12p13.1 | CDKN1B-1 | 0.79-1.23 | 0.68-1.34 |
| 17p13.1 | TP53-10 | 0.94-1.06 | 0.91-1.09 |
| 17p13.1 | TP53-4b | 0.80-1.32 | 0.67-1.45 |
| 17p13.1 | TP53-1 | 0.91-1.07 | 0.87-1.11 |
| 17q11.2 | NF1-17 | 0.89-1.09 | 0.84-1.14 |
| 17q11.2 | SUZ12-12 | 0.82-1.18 | 0.73-1.27 |
| 17q12 | AATF-11 | 0.84-1.10 | 0.78-1.16 |
| 19p13.2 | SMARCA4-25 | 0.72-1.28 | 0.58-1.42 |
| 19q13.42 | PRPF31-14 | 0.81-1.21 | 0.71-1.31 |
| 20q11.21 | ASXL1-4 | 0.82-1.22 | 0.72-1.32 |
| 20q11.23 | SRC-6 | 0.88-1.16 | 0.81-1.23 |
| 20q13.12 | MMP9-9 | 0.78-1.26 | 0.66-1.38 |
| 20q13.12 | ZMYND8-14 | 0.90-1.10 | 0.85-1.15 |

Sup. Table2 The 112 genes sequenced in this study

| ABCB1 | ABL1 | ADAMTS13 | AKT1 | ALAS2 | ARID1A | ASXL1 | ATM |
| --- | --- | --- | --- | --- | --- | --- | --- |
| BCL2 | BCL6 | BIRC3 | BRAF | CALR | CBL | CCND1 | CCND3 |
| CDKN1A | CEBPA | c-MAF | c-MYC | CREBBP | CRLF2 | CSF3R | CUX1 |
| CXCR4 | CYLD | DDX3X | DIS3 | DNM2 | DNMT3A | ECT2L | EED |
| EGFR | EP300 | EPHA7 | EZH2 | FAM46C | FANCA | FANCC | FANCG |
| FAT1 | FBXW7 | FGFR3 | FLT3 | GATA2 | GATA3 | IDH1 | IDH2 |
| IL7R | ITK | JAK1 | JAK2 | JAK3 | KIT | KRAS | SH2B3 |
| LYST | MAFB | MAPK1 | MLL2 | MPL | MUM1 | MYD88 | MYH11 |
| NF1 | NOTCH1 | NOTCH2 | NPM1 | NRAS | PAX5 | PDGFRB | PHF6 |
| PIK3CA | PRDM1 | PRF1 | PRMT5 | PRPF40B | PTEN | PTPN11 | RAB27A |
| RB1 | RELN | RUNX1 | SAMHD1 | SETBP1 | SF1 | SF3A1 | SF3B1 |
| SH2D1A | SMC1A | SMC3 | SRSF2 | STX11 | STXBP2 | SUZ12 | TAL1 |
| TET2 | TP53 | TEL/ETV6 | TRAF3 | U2AF1 | U2AF2 | UNC13D | WAS |
| WHSC1 | WT1 | TNFAIP3/A20 | XIAP | XPO1 | ZMYM3 | ZRSR2 | IKZF1 |

Sup. Figure 1. The abnormalities detected by MLPA.(A) Normal karyotype. (B) MLPA detection of -7 in a patient with normal G-band karyotype. (C) MLPA detection of +8/11q+ in a patient with normal G-band karyotype. (D) MLPA detection of 5q-/+8 in a patient with 47,XY,+8[20].

Figure 2. Overall survival of normal karyotype IPSS-R lower risk patients with CNVs detected by MLPA (median OS: not reached) and patients without CNVs (median OS: not reached).
